# *Acinetobacter baumannii* biofilm biomass mediates tolerance to cold plasma

**DOI:** 10.1111/lam.13122

**Published:** 2019-03-13

**Authors:** P.B. Flynn, W.G. Graham, B.F. Gilmore

**Affiliations:** ^1^ Biofilm Research Group School of Pharmacy Queen's University Belfast UK; ^2^ Centre for Plasma Physics (CPP) School of Mathematics and Physics Queens University Belfast UK

**Keywords:** antimicrobials, biofilms, biocides, disinfection, nonthermal processes

## Abstract

*Acinetobacter baumannii* is an intrinsically multidrug‐resistant pathogen that, when existing as a biofilm, confers increased environmental tolerance to desiccation, nutrient starvation as well as increased tolerance to antimicrobials. Outbreaks of *A. baumannii* infections within the clinical setting are often associated with the biofilm phenotype. This study investigates the role of biofilm biomass in *A. baumannii* susceptibility to exposure to a kilohertz‐driven, in‐house–designed, cold plasma jet, through the examination of cold plasma treatment efficacy in *A. baumannii* biofilms grown over various times for up to 72 h. For biofilms grown for 24, 48 and 72 h, *D* values were 19·32 ± 2·71, 29·18 ± 3·15 and 24·70 ± 3·07 s respectively. Monitoring *A. baumannii* biofilm biomass over these time periods revealed that the greatest biomass was observed at 48 h with the lowest biofilm biomass at 24 h growth. Enumeration of viable biofilm colony counts at each time point was comparable. Scanning electron microscopy images of plasma‐treated biofilms revealed extensive surface damage of *A. baumannii* cells. These results describe the role of biomass in mediating *A. baumannii* biofilm susceptibility to cold plasma treatment, implicating the biofilm matrix as a protective barrier to the antimicrobial effects of cold plasma.

**Significance and Impact of the Study:**

*Acinetobacter baumannii* biofilm formation results in increased environmental and antimicrobial tolerance and resistance compared to the planktonic phenotype. Cold plasma technology is increasingly investigated as a new tool for decontamination of biofilm‐contaminated surfaces, especially those found in the clinical setting. This new technology presents a promising approach to the remediation of surfaces contaminated by biofilms. This study identifies the role played by *A. baumannii* biofilm biomass in mediating tolerance and susceptibility to cold plasma treatment. This work demonstrates that increased biofilm biomass reduces the efficacy of antimicrobial species generated by cold plasma, resulting in greater tolerance to plasma exposure.

## Introduction

*Acinetobacter baumannii* is a Gram‐negative coccobacillus which is part of the ‘ESKAPE’ pathogens group, identified as the most problematic causative agents of human infection, due to increasing incidence of antibiotic resistance (Boucher *et al*. 2009). In addition, it has recently been listed as a top priority by the WHO for the development of new antibiotics (WHO, 2017). *Acinetobacter baumannii* is most often encountered in intensive care units (ICUs) where it accounts for up to 20% of ICU infections worldwide and is a problematic pathogen due to production of a range of virulence factors, including rapid development of multidrug resistance and formation of highly antimicrobial‐tolerant biofilms (Vincent *et al*. 2009; Joshi 2013). Biofilm formation of *A. baumannii* contributes to its exceptional environmental persistence, aiding its survival for months on surfaces and its persistence in dry and nutrient‐deprived conditions (Orsinger‐Jacobsen *et al*. 2013; Gayoso *et al*. 2014; Chapartegui‐González *et al*. 2018). Its ability to form biofilms on a range of surfaces in unfavourable conditions renders the biofilm phenotype an important virulence factor in *A. baumannii* infections.

Atmospheric pressure non thermal plasmas or, more simply, cold plasmas are an innovative and nascent technology for the disinfection and decontamination of microbial biofilms on abiotic and biotic surfaces (Gilmore *et al*. 2018). Cold plasmas are partially ionized gases (typically using air, helium or argon) that can be produced from the input of electrical energy into a gas, generating a plasma at or near ambient temperatures. The antimicrobial activity cold plasma relies on the production of electrical currents, charged species, UV light and on the production of reactive oxygen and nitrogen species (RONS), such as hydrogen peroxide, nitrite, nitrates, ozone, hydroxyl radical and peroxynitrite anion, at atmospheric pressure and at biologically relevant temperatures. Previously it was reported that cold plasma treatment of both planktonic and biofilms from the ESKAPE cohort of pathogens resulted in complete eradication (>6 log reduction) in under 6 min of treatment with the exception of *A. baumannii* biofilms, whereby only ≥4 log reduction was achieved in this time (Flynn *et al*. 2015). Based on the previously reported elevated tolerance of *A. baumannii* to cold plasma exposure, this study aims to investigate how *A. baumannii* biofilm growth time and biomass influence susceptibility to cold plasma.

## Results and discussion

*Acinetobacter baumannii* biofilms were grown over several time periods (resulting in significant changes in their biomass) and exposed to a kHz driven, helium/oxygen cold plasma jet. Biofilms were grown for 24, 48 and 72 h in order to gain an insight into how biomass changes can alter the inactivation kinetics of *A. baumannii* biofilms when exposed to nonthermal plasma.

**Figure 1:**
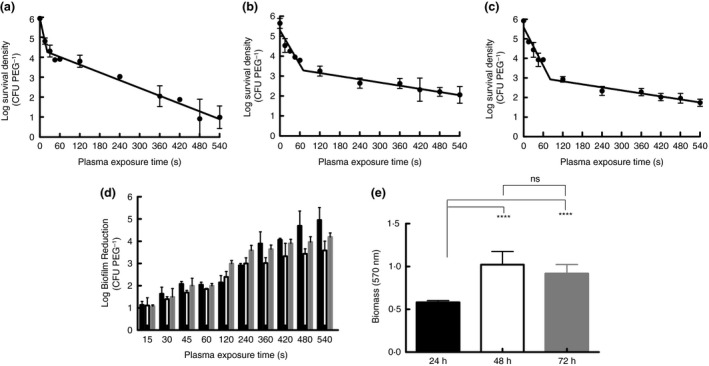
Survival curves for *Acinetobacter* b*aumannii* biofilms grown for (a) 24 h, (b) 48 h and (c) 72 h respectively. (d) Exhibits log reduction of viable for biofilms grown for 24 h (black bars), 48 h (white bars) and 72 h (grey bars). Each bar represents the mean of three replicates with the standard error of the mean. (e) Demonstrates biomass of *A. baumannii* biofilms grown for 24 h (black bars), 48 h (white bars) and 72 h (grey bars). Each bar represents the mean of 12 replicates with error bars representing the standard error of the mean. One‐way anova with Tukey's multiple comparisons test performed. ****P value < 0·0001.

In order to assess the effect of biofilm growth time on *A. baumannii* tolerance to plasma exposure (as a function of biofilm biomass), *A. baumannii* biofilms were grown using the Calgary Biofilm Device. This device is commonly used for the MBEC High Throughput (HTP) Assay for antimicrobial efficacy against biofilms, although in this case it was amended in order to evaluate plasmas' antibiofilm activity as described in previous studies (Alkawareek *et al*. 2012a,b, 2014a; Alshraiedeh *et al*. 2016). Figure 1a–c displays survival curves for *A. baumannii* biofilms following plasma exposure for up to 9 min. *A. baumannii* biofilms were not completely eradicated, even after 9 min of exposure for any of the three growth times. The bi‐phasic kill curves are commonly observed with plasma exposure of biofilms, and are attributed to the reactive oxygen and RONS generated by cold plasmas damaging and inactivating superficial bacterial cells on the periphery of the biofilm. This accounts for the rapid initial kill, followed by a ‘tailing’ or slowing of the kill rate that may be due to either; the build‐up of cellular debris/aggregates decreasing the accessibility of, or sequestering, the active species of cold plasma to bacterial cells, desiccation or potentially the development of a persister phenotype (the presence of a subpopulation of highly stressed tolerant cells). As to whether the lack of complete eradication even over extended exposure times is due to plasma exposure driving the emergence of a subpopulation of cells induced into a plasma tolerant‐like (persister) state or simply due to the presence of cellular debris restricting the antimicrobial species generated by the cold plasma, remains to be elucidated. Decimal reduction times or *D* values were calculated from the negative reciprocal of each bi‐phasic kill curve and are shown in Table 1. There was a significant difference between 48 and 24 h biofilms’ *D* values for both phases of the time‐kill curve. Figure 1d presents a direct comparison of the log reduction in each biofilm relative to the control. It must also be noted that each biofilm control peg had comparable log viable cell counts at 0 s exposure on Fig. 1a–c, with no significant differences in the viable microbial load. Crystal violet staining of biofilms is an indiscriminate method of quantification which includes all components of a biofilms biomass, not only viable cells. The 48 h biofilms had the greatest biomass, while also exhibiting the greatest tolerance to plasma (based on D values from Table 1 and lowest log reduction of viable cells from Fig. 1d). This finding is corroborated by Alshraiedeh *et al*. (2016) who observed that increasing biofilm biomass in a range of *Burkholderia cepacia* clinical isolate strains correlated with increased plasma tolerance. The difference in biomass would most likely be due to differences in the amount of biofilm matrix and extracellular polymeric substances (EPS), given that viable counts in each biofilm (24, 48 and 72 h) are comparable. It has been demonstrated previously that cell titre plays a role in plasma inactivation of bacteria (Laroussi *et al*. 2011), however, in this case there were no significant changes in viable cell count over each growth period, only changes in biomass. This allowed comparison of the same bacterial strain with differing biomass being the independent variable. The biomass in this instance takes into account biofilm components and not just bacterial cells. Experiments by Fernández *et al*. (2012) described the protective effect cell biomass has on viable cells. Cellular biomass was provided by heat treated *Salmonella enterica* serovar Typhi cells which would not entirely reflect a biofilm matrix or EPS (Flemming and Wingender 2010). In addition to the biofilm matrix, dead, lysed cells and their cellular components will be present during plasma exposure of biofilms and would contribute to the phasic nature of survival curves (Morent and Geyter 2011; Alkawareek *et al*. 2012b), via attenuation the biocidal activity of plasma‐produced reactive species, in a similar fashion to exogenous organic matter attenuation of the disinfectant activity of conventional biocides.

**Table 1:** Decimal reduction times (*D* value) for plasma‐exposed *Acinetobacter* b*aumannii* biofilms grown for 24, 48 and 72 h. Each *D* value represents the average of three replicates with the standard error of the mean (SE) statistical significance of Dunn's multiple comparisons test shown with a indicating significance between 48 h and 24 h

	24 h	48 h	72 h
D1 value (seconds **±** SE)	19·32 ± 2·71	29·18 ± 3·15^a^	24·70 ± 3·07
D2 value (seconds **±** SE)	169·97 ± 26·27	299·16 ± 42·15^a^	242·15 ± 15·81

a *P* value < 0·05.

**Figure 2:**
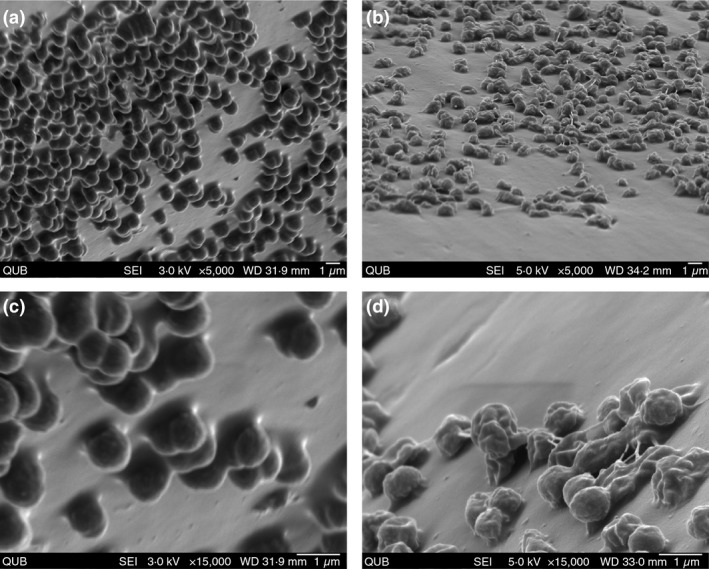
Scanning electron microscopy images (SEM) of an unexposed 24‐h *Acinetobacter* b*aumannii* biofilm (a) and (c) and a 6‐min, plasma‐treated *A. baumannii* biofilm (b) and (d) captured using a JEOL field emission SEM JSM‐6500F.

Scanning electron microscopy (SEM) analysis of *A. baumannii* biofilms exposed to plasma was performed in order to visually inspect the effect plasma had on biofilm cells. Figure 2 shows unexposed biofilms alongside 360 s plasma‐treated *A. baumannii* respectively. These high‐resolution SEM images provide a clear appreciation of an untreated biofilm with (a) and (c) in Fig. 2. These images show microcolonies (discrete matrix enclosed cell stacks) and cellular appendages between the cells and surface, a characteristic of biofilms (Burmølle *et al*. 2010). *A. baumannii* biofilms were imaged using SEM after being exposed for 360 s. Compared to the control biofilms, there is an observable change in cellular morphology postexposure. *A. baumannii* cells after plasma exposure have lost their cellular integrity, becoming flat amorphous cells with indentions on the cell surface. This is a common observation with plasma treatment of bacterial cells. Xu *et al*. (2011) and Cahill *et al*. (2014) used transmission electron microscopy (TEM) and atomic force microscopy (AFM), respectively, to inspect damage to the cell surface with altered morphology for both Gram‐negative and Gram‐positive bacteria. This implicates the cell surface as a primary site for plasma‐mediated damage. A mechanism of action which includes plasma‐mediated direct physical damage to bacterial cells is supported by previous studies which have employed cell membrane biomarkers to interrogate plasma‐mediated cell membrane damage. Malondialdehyde, a product of lipid peroxidation, has been shown to be produced on plasma exposure of *Escherichia coli* cells (Alkawareek *et al*. 2014b) and Kvam *et al*. (2012) demonstrated a rapid decline in intracellular ATP activity of bacterial cells after plasma exposure.

This study highlights the important role of biomass in *A. baumannii* biofilm tolerance to cold plasma exposure. From these findings, further insights into whether tolerance can be attributed to a physical mechanism implicating cell debris and biofilm components or the identification of a persistent subpopulation is required.

## Materials and methods

### Bacterial strains and growth conditions

*Acinetobacter baumannii* NCTC 13304 was stored at −20°C in Microbank vials (Pro – Lab Diagnostics, Cheshire, UK) and subcultured overnight at 37°C on Muller–Hinton agar (MHA) (Oxoid Ltd., Basingstoke, UK).

### Growth of *A. baumannii* biofilms using the Calgary Biofilm Device (CBD)

Biofilms of *A. baumannii* NCTC 13304 were grown using the Calgary Biofilm Device (CBD) (Innovotech Inc., Edmonton, Alberta, Canada). *A. baumannii* was grown overnight on Muller–Hinton Agar (MHA) at 37°C. Colonies of similar size and morphology were used to inoculate Muller–Hinton Broth (MHB) to an OD_550_ equivalent to 1 × 10^7^ CFU per ml. One hundred and fifty microlitres of this inoculum was transferred into each well of the CBD. Biofilms were grown for 24 h, 48 h and 72 h at 37°C in an orbital incubator (100 rev min^−1^) with fresh MHB supplied every 24 h for each corresponding growth time. At the end of each incubation time, for plasma‐exposed biofilms, the CBD pegs were removed from the lid using flamed pliers and gently rinsed twice in 150 *μ*l of phosphate buffer saline (PBS).

### Biofilm exposure to cold plasma and surviving cell enumeration

Biofilms were exposed to the kHz‐driven helium/oxygen cold plasma for 0, 15, 30, 45, 60, 120, 240, 360, 420, 480 and 540 s. Details and characteristics of the plasma jet have previously been described (Alkawareek *et al*. 2012b; Flynn *et al*. 2016). In this study, the powered electrode was positioned 10 mm from the end of the quartz tube and the interelectrode distance between the powered and grounded electrode was 25 mm. Pegs of the CBD were placed upright at a 15‐mm distance from the top of the peg to the end of the nozzle as described in (Alkawareek *et al*. 2012b). The cold plasma jet produces a plume which is used to treat the biofilms in air. After plasma exposure pegs were placed in 200 *μ*l of PBS and sonicated for 15 min using a Branson 3510 ultrasonic cleaner (40 kHz, 130–335 W (Branson, Danbury, CT). Serial 10‐fold dilutions of sonicated biofilm suspensions were prepared, and three 20 *μ*l drops from each dilution were plated onto MHA for standard plate counts. Plates were incubated aerobically at 37°C and observed after 24 and 48 h for viable colonies which were used to create log survival curves. Log reduction, calculated by subtracting the log surviving colonies from the log of unexposed controls, was used to compare between growth time susceptibility as well calculation of the *D* value from log survival curves.

### Measurement of *A. baumannii* biofilm biomass

Crystal violet (CV) staining was employed to ascertain biofilm biomass of 24, 48 and 72 h biofilms. Using 12 biofilm pegs for each growth time, pegs were gently rinsed twice in 150 *μ*l of PBS before staining in 150 *μ*l 0·1% crystal violet for 15 min. After which pegs were gently rinsed twice again with 200 *μ*l of PBS to remove excess CV. The pegs were left to dry at room temperature for 24 h. For biomass measurements the retained crystal violet on the biofilm was resolubilized with 30% v/v acetic acid: water and read on a plate reader (BioTek EL808) at 570 nm.

### Scanning Electron Microscopy (SEM) of plasma‐treated *A. baummannii* biofilms

Preparation of biofilms for SEM was completed based on the MBEC SEM procedure with minor adjustments as follows: Two pegs for plasma exposure and untreated pegs, were randomly chosen and gently removed from the CBD lid. The removed pegs were then rinsed with 150 *μ*l of PBS for 1 min to remove planktonic and loosely adherent bacteria and exposed to plasma for 120 and 360 s. Plasma exposed and untreated pegs were fixed in separate 1·5‐ml Eppendorf tubes containing 1 ml of 3% glutaraldehyde in 0·1 mol l^−1^ cacodylic acid (pH 7·2) overnight, at 4°C. After fixation, pegs were washed four times for 30 min with 250 *μ*l of 0·1 mol l^−1^ cacodylate buffer at 4°C. Immediately after the wash, samples were dehydrated at room temperature in five steps by placing the pegs for 30 min in 250 *μ*l of 50, 70 and 90% and then twice for 30 min in 100% ethanol. The pegs were then transferred to a microtitre plate containing 200 *μ*l of hexamethyldisilazane (HMDS) and left to dry for 24 h in the fume cupboard. The specimens were mounted on aluminium stubs using Araldite^®^ epoxy resin, sputter coated in pure gold and examined using the field emission scanning electron microscope FE‐SEM (JEOL JSM‐6500F FE‐SEM, JEOL Ltd., Japan). SEM images were recorded at 5000× and 15 000× magnifications with at least two images recorded at each magnification.

### Statistical analysis

Statistical analyses were performed using GraphPad Prism 6 (GraphPad Software Inc., San Diego, CA). The means and standard deviations for the CV biomass assay were calculated based on 12 replicates. Parametric one‐way analysis of variance (anova) was used to analyse statistical differences with Tukey's multiple comparisons test analysis used to determine statistical differences between CV biomass grown for 24, 48 and 72 h. Data were shown to be normally distributed using the D'Agostino & Pearson normality test. In all cases, a probability of *P* < 0·0001 (****) denoted significance. Decimal reduction times were calculated from the negative reciprocal of the slope of bi‐phasic survival curves and analysed using Kruskal–Wallis test with Dunn's multiple comparisons test. * indicates a *P* < 0·05.

## Conflict of Interest

The authors declare no conflict of interest.
